# Supplementary figures and images for: A pyroptosis gene-based prognostic model for predicting survival in low-grade glioma
Source: PeerJ. 2023 Nov 13;11:e16412. doi: 10.7717/peerj.16412 (PMC10652862; doi:10.7717/peerj.16412)

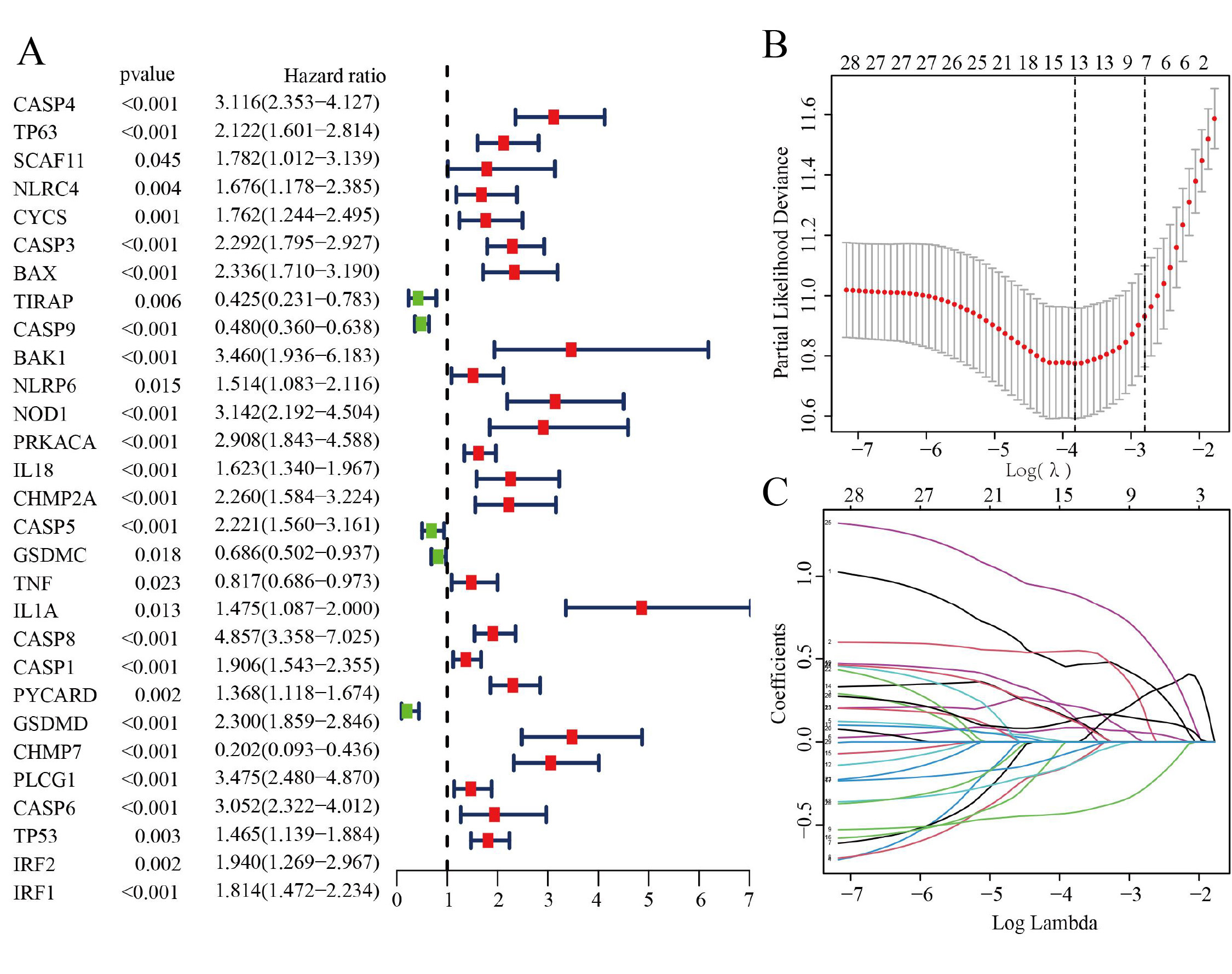

Supplement: Supplemental Information 1 — (A) Univariate Cox regression analysis of OS for 29 prognostic genes. Five prognostic genes (HR <1) were protective genes, and 24 prognostic genes (HR¿1) were risk genes. (P < 0.05). (B) LASSO analysis was used to screen genes with high correlation. The horizontal axis indicates the logarithm of the independent variable lambda, and the vertical axis indicates the error of cross-validation. (C) Tuning parameter (λ ) selection cross-validation error curve for LASSO analysis. [file peerj-11-16412-s001.jpg]

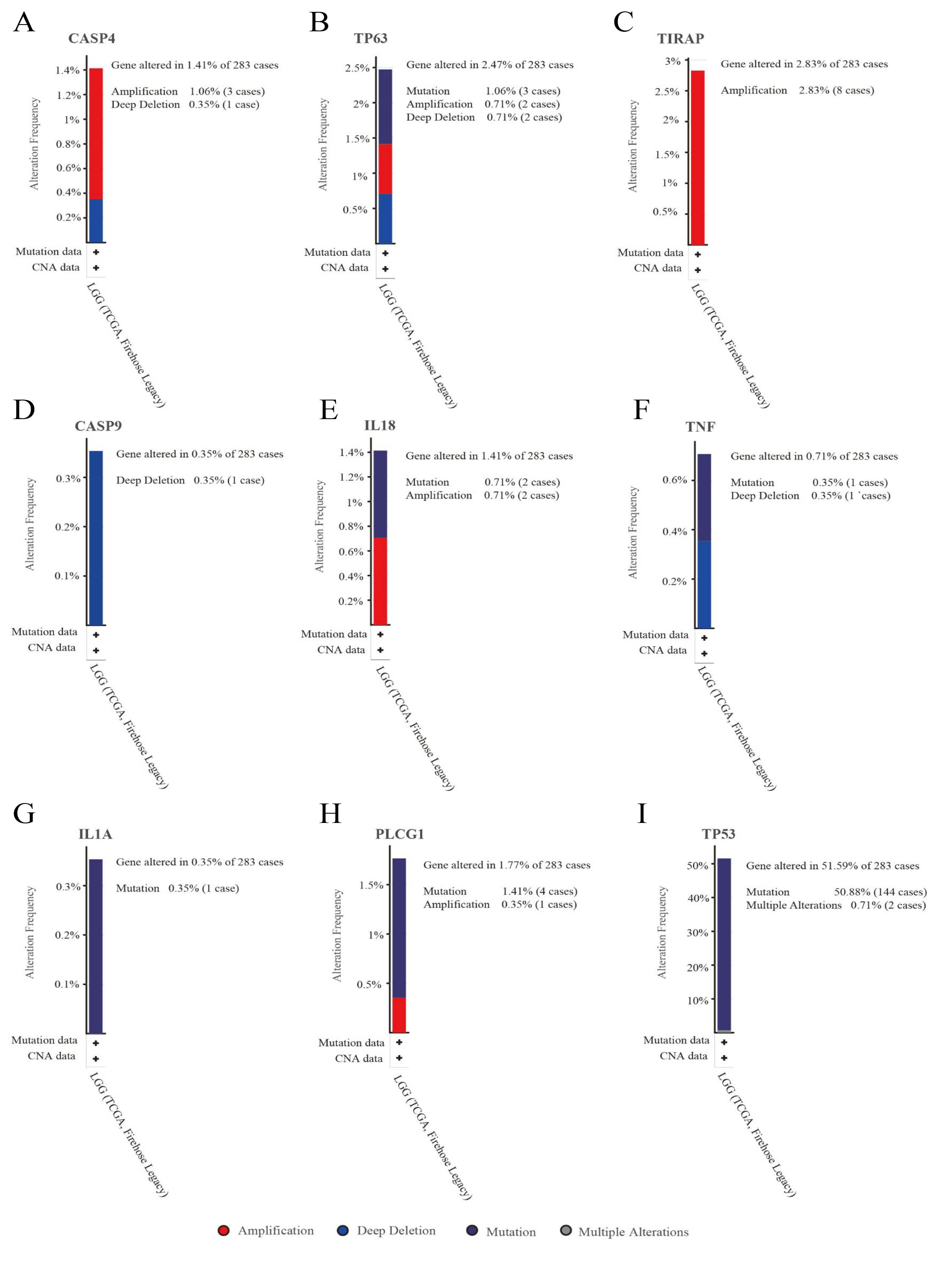

Supplement: Supplemental Information 2 — (A) The CNV status of CASP4. (B) The CNV status of TP63. (C) The CNV status of TIRAP. (D) The CNV status of CASP9. (E) The CNV status of IL18. (F) The CNV status of TNF. (G) The CNV status of IL1A. (H) The CNV status of PLCG1. (I) The CNV status of TP53. [file peerj-11-16412-s002.jpg]
